# Alizarin Red S-Confined Layer-By-Layer Films as Redox-Active Coatings on Electrodes for the Voltammetric Determination of L-Dopa

**DOI:** 10.3390/ma10060581

**Published:** 2017-05-25

**Authors:** Shigehiro Takahashi, Iwao Suzuki, Tatsuro Sugawara, Masaru Seno, Daichi Minaki, Jun-Ichi Anzai

**Affiliations:** 1 Faculty of Pharmacy, Takasaki University of Health and Welfare, 37-1 Nakaorui, Takasaki 370-0033, Japan; takahashi-shi@takasaki-u.ac.jp (S.T.); suzuki@takasaki-u.ac.jp (I.S.); 2 Graduate School of Pharmaceutical Sciences, Tohoku University, Aramaki, Aoba-ku, Sendai 980-8578, Japan; b5yd1006@dc.tohoku.ac.jp (T.S.); b4ym1018@s.tohoku.ac.jp (M.S.); b3yb1047@s.tohoku.ac.jp (D.M.)

**Keywords:** layer-by-layer film, electrode, voltammetry, alizarin red S, L-dopa

## Abstract

The preparation of redox-active coatings is a key step in fabricating electrochemical biosensors. To this goal, a variety of coating materials have been used in combination with redox-active compounds. In this study, alizarin red S (ARS) was confined in layer-by-layer (LbL) films composed of poly(ethyleneimine) (PEI) and carboxymethylcellulose (CMC) to study the redox properties. A gold (Au) disc electrode coated with PEI/CMC LbL film was immersed in an ARS solution to uptake ARS into the film. ARS was successfully confined in the LbL film through electrostatic interactions. The cyclic voltammogram (CV) of ARS-confined PEI/CMC film-coated electrodes thus prepared exhibited redox waves in the potential range from −0.5 to −0.7 V originating from 9,10-anthraquinone moiety in ARS, demonstrating that ARS preserves its redox activity in the LbL film. An additional oxidation peak appeared around −0.4 V in the CV recorded in the solution containing phenylboronic acid (PBA), due to the formation of a boronate ester of ARS (ARS-PBA) in the film. The oxidation peak current at −0.4 V decreased upon addition of 3,4-dihydroxyphenylalanine (L-dopa) to the solution. Thus, the results suggest a potential use of the ARS-confined PEI/CMC films for constructing voltammetric sensors for L-dopa.

## 1. Introduction

Layer-by-layer (LbL) polyelectrolyte films have been widely used for the development of biosensors [1,2], surface coatings [3,4,5], drug delivery systems [6], and so forth. LbL films are prepared by the alternate deposition of oppositely-charged polyelectrolytes on the surface of solid substrates through electrostatic interactions. Synthetic polymers such as poly(carboxylate)s and poly(sulfonate)s are typical polyanions used, while poly(amine)s are frequently used as cationic counterparts [7]. Synthetic polymers provide excellent chemical and mechanical stabilities of LbL films. Therefore, synthetic polymer-based LbL films are useful for developing robust surface coatings. In addition, biopolymers including polysaccharides, proteins, and DNA are also available for preparing LbL films because these biopolymers contain positive or negative charges depending on the pH of the medium. An advantage of biopolymer-based LbL films is that bio-functionalities such as catalysis and molecular recognition can be introduced in the films. For example, enzyme-containing LbL films have been utilized for preparing electrochemical biosensors [8,9]. 

Recently, much attention has been devoted to the development of redox-active LbL films because of their potential applications to electrochemical devices such as sensors and releasing systems [10,11]. LbL films are redox inactive as long as conventional polymers and biopolymers are employed as film components. In contrast, redox-active LbL films can be prepared by immobilizing redox-active compounds in LbL films. Two different routes are employed for constructing redox-active LbL films; the use of redox-active polymers as a film component and/or the modification of as-prepared LbL films with redox-active compounds. The former route provides redox-active LbL films with high stability because of the covalent linkage of redox-active moieties to polymer chains. The latter route, on the other hand, provides a facile protocol for preparing redox-active LbL films without synthesizing redox-active polymers. Typically, redox-active compounds are immobilized in LbL films through electrostatic interactions by immersing LbL films in a solution of redox-active compound. In this protocol, the loading of redox-active compounds in LbL films can be regulated by changing variables such as the pH and ionic strength of the solution. In the present study, we prepared alizarin red S (ARS)-confined LbL films on the surface of a gold (Au) electrode to study the voltammetric properties. 

**Figure 1:**
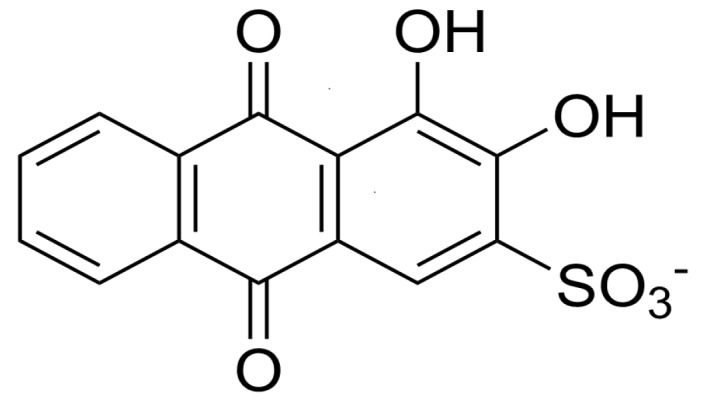
Chemical structure of ARS.

ARS has been used as an electrochemical indicator for the determination of ions and molecules by taking advantage of the redox-active nature of the 9,10-anthraquinone moiety in ARS [12,13,14,15] (Figure 1). For example, the cyclic voltammogram (CV) of ARS significantly changed upon binding Ca^2+^ ions to the 1,2-dihydroxy group, enabling the voltammetric determination of Ca^2+^ ions [15]. In addition, phenylboronic acid (PBA) ester of ARS (ARS-PBA) can be used in a voltammetric displacement assay, in which voltammetric signals arising from ARS-PBA decreased upon adding diol compounds to the sample solutions [16,17,18,19]. In these studies, ARS-PBA was dissolved in a solution [16] or adsorbed onto electrodes by using polymers and carbon nanomaterials as supports [17,18,19]. Thus, ARS-PBA-modified electrodes are useful for the development of electrochemical devices. However, the reported procedures include synthesis and derivatization of materials before electrode modification, which are somewhat tedious. 

We report here a facile protocol for the preparation of ARS-immobilized LbL films and their use for the electrochemical determination of L-dopa. To this goal, we used an Au disc electrode coated with LbL films composed of poly(ethyleneimine) (PEI) and carboxymethylcellulose (CMC). The PEI/CMC film-coated electrode was immersed in an ARS solution, by which ARS was electrostatically confined in the LbL film. The ARS-confined PEI/CMC film-coated electrodes thus prepared exhibited a pair of redox peaks in the CV, showing that ARS is redox active even in the LbL films. Furthermore, a new oxidation peak was observed in the CV recorded in a PBA solution, due to the formation of an ARS-PBA conjugate in the films. Interestingly, the intensity of the oxidation peak depended on the concentrations of L-dopa added to the solution, suggesting a potential use of the ARS-confined electrode as a voltammetric sensor for the determination of L-dopa. 

## 2. Materials and Methods 

### 2.1. Reagents

Sodium 3-mercapto-1-propanesulfonate (MPS) was purchased from Sigma-Aldrich Chemical Co. (St. Louis, MO, USA). PEI (30% aqueous solution, molecular weight (MW): 60,000–80,000) and CMC sodium salt (MW: ~250,000) were obtained from Nacalai Tesque Co. (Kyoto, Japan) and Tokyo Kasei Co. (Tokyo, Japan), respectively. PEI has a random branched structure, with the ratio of primary, secondary, and tertiary amino groups being nominally about 1:2:1. ARS and L-dopa were from Nacalai Tesque Co. All chemicals were of reagent grade and were used without further purification. The Au disc electrode (diameter, 3 mm) was purchased from BAS Co. (Tokyo, Japan). 

### 2.2. Preparation of ARS-Confined LbL Films

LbL films were prepared on a quartz slide (50 × 9 × 1 mm^3^), that had been cleaned with a sulfuric acid/chromic acid mixture, to study the binding of ARS spectroscopically. LbL films composed of PEI and CMC were coated on the surface of the quartz slide by alternately immersing the slide in 0.1 mg·mL^−1^ PEI and 0.1 mg·mL^−1^ CMC solutions (10 mM 4-(2-hydroxyethyl)-1-piperazineethanesulfonic acid (HEPES) buffer containing 100 mM NaCl, pH 7.5) for 15 min, according to the reported procedure [20]. The quartz slide was rinsed in distilled water for 5 min after each deposition. The deposition was repeated to prepare 5.5 and 10.5-bilayer LbL films. The LbL film-coated slide was then immersed in a 0.1 mM ARS solution in the 10 mM HEPES buffer for 2 h to uptake ARS from the solution into the films, followed by immersing the slide in the buffer solution overnight to remove weakly bound ARS. The adsorption of ARS to the film was evaluated by recording UV-visible absorption spectra of the film-coated slide. 

In a similar manner, LbL films composed of PEI and CMC were coated on the surface of the Au disc electrode, which had been polished with alumina slurry and thoroughly rinsed in deionized water. Prior to modification, the surface of the electrode was cleaned by scanning an electrode potential from −0.2 to +1.5 V in the scan rate of 0.1 V s^−1^ in 0.5 M H_2_SO_4_. The electrode surface was first modified with a self-assembled MPS monolayer by immersing the electrode in an aqueous MPS solution (10 mM) overnight. Then, PEI and CMC were alternately deposited to form the LbL films on the surface of the electrode. The LbL film-coated Au electrode was immersed in a 0.1 mM ARS solution to uptake ARS into the films, and then rinsed in a similar manner.

### 2.3. Atomic Force Microscopy (AFM)

AFM (SPM-9600, Shimadzu Co., Kyoto, Japan) was used to evaluate the thickness of the films. LbL films were prepared on a glass slide (diameter 15 mm). The film-coated slide was rinsed in pure water and dried for 24 h in a desiccator. Film thickness was determined by scratching the film-coated glass slide with a blade and scanning over the scratch in contact mode at room temperature in air. 

### 2.4. Electrochemical Measurements

CV and differential pulse voltammogram (DPV) of LbL film-coated electrodes were recorded using a conventional three-electrode system with a platinum wire counter electrode and a silver/silver chloride (Ag/AgCl) reference electrode. All measurements were performed under a nitrogen (N_2_) atmosphere after the solution was purged with N_2_ gas. All measurements were carried out in 10 mM HEPES buffer containing 10 mM NaCl at an ambient temperature (ca. 23 °C). 

## 3. Results and Discussion

### 3.1. Immobilization of ARS in PEI/CMC LbL Films

**Figure 2:**
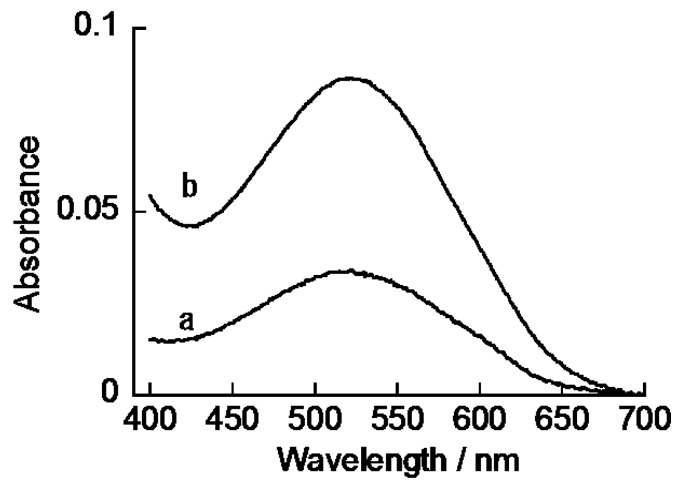
UV-visible spectra of ARS-immobilized (PEI/CMC)_5_PEI (**a**) and (PEI/CMC)_10_PEI films (**b**). The spectra were recorded by immersing the LbL film-coated quartz slides in 10 mM HEPES buffer (pH 7.5).

Polyelectrolyte LbL films are often used as scaffold to immobilize small ions and molecules for constructing functional surface coatings [21]. In this context, it may be possible to immobilize ARS in PEI/CMC LbL films through an electrostatic affinity between negatively-charged ARS and positive charges in PEI/CMC films, because PEI/CMC films contain excess positive charges originating from PEI chains. In this context, we reported that negatively-charged [Fe(CN)_6_]^3−/4−^ ions were confined in the PEI/CMC films through an electrostatic force of attraction [20]. It is known that ARS assumes a dianionic form at neutral pH, in which 2-hydroxy and 3-sulfonate groups are dissociated [22]. Therefore, it is anticipated that ARS is confined in PEI/CMC LbL films through electrostatic interactions. 

(PEI/CMC)_5_PEI and (PEI/CMC)_10_PEI films were prepared on the surface of a quartz slide to evaluate the binding property of ARS to the films by means of UV-visible absorption spectroscopy. Figure 2 shows the UV-visible absorption spectra of (PEI/CMC)_5_PEI and (PEI/CMC)_10_PEI LbL films after treatment in 0.1 mM ARS solution at pH 7.5. The LbL films exhibited no absorption band in the range of 400–700 nm before ARS treatment. In contrast, the films exhibited an absorption band at 510 nm after being treated in the ARS solution followed by thorough rinsing, which suggests that ARS was immobilized in the LbL films. The release of ARS from the films into solution was negligible, showing that ARS was irreversibly confined in the films. The loadings of ARS in the (PEI/CMC)_5_PEI and (PEI/CMC)_10_PEI films are calculated to be 2.7 × 10^−9^ and 6.8 × 10^−9^ mol·cm^−2^, respectively, from the UV-visible absorption spectra. We have evaluated the thickness of the (PEI/CMC)_5_PEI and (PEI/CMC)_10_PEI films using AFM to be approximately 35 and 75 nm, respectively. Therefore, the contents of ARS in the films are calculated to be 7.7 × 10^−4^ and 9.1 × 10^−4^ mol cm^−3^, respectively. Thus, the concentration of ARS in the films was roughly comparable to each other, irrespective of the film thickness, suggesting that ARS bound throughout the films not only to the outermost PEI layer. We have studied the effect of pH on the ARS binding and found that the amount of immobilized ARS in the (PEI/CMC)_10_PEI film increased in the order of 6.4 × 10^−9^ mol·cm^−2^ (pH 8.5) < 6.8 × 10^−9^ mol cm^−2^ (pH 7.5) < 8.0 × 10^−9^ mol·cm^−2^ (pH 6.5). The ARS contents at pH 6.5–8.5 were estimated from the absorption spectra, assuming that ARS exists in its dianionic form in this pH range [22]. The pH-dependent binding of ARS may relate to the fact that the amount of net excess charge in the film originating from PEI chains increases with the decreasing pH of the media. In other words, the number of binding sites for ARS in the films increases in the order of pH 8.5 < pH 7.5 < pH 6.5.

### 3.2. Redox Properties of ARS in PEI/CMC LbL Films

**Figure 3:**
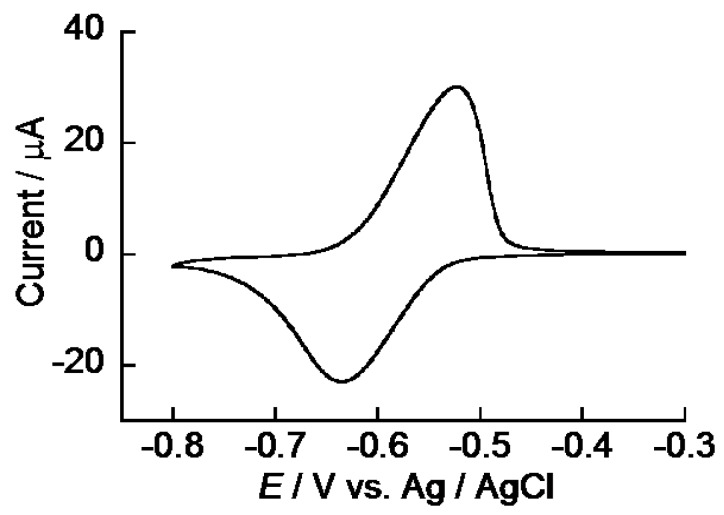
Typical CV of the Au electrode coated with ARS-immobilized (PEI/CMC)_10_PEI film recorded in 10 mM HEPES buffer at pH 7.5. Scan rate, 50 mV·s^−1^.

**Figure 4:**
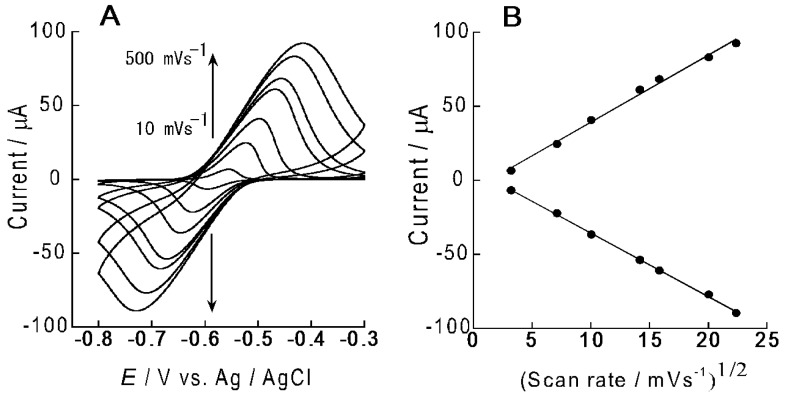
(**A**) The effect of scan rate on the CV of the ARS-immobilized (PEI/CMC)_10_PEI film-coated Au electrode in 10 mM HEPES buffer at pH 7.5; (**B**) Plots of redox peak currents of the CV vs. a square root of the scan rate.

Redox properties of ARS in the (PEI/CMC)_10_PEI LbL film were studied with CV using the ARS-immobilized (PEI/CMC)_10_PEI film-coated electrode. (PEI/CMC)_10_PEI films were deposited on the surface of the Au electrode by alternatingly immersing the electrode in 0.1 mg·mL^−1^ PEI and CMC solutions at pH 7.5. Then, the (PEI/CMC)_10_PEI film-coated electrode was immersed in 0.1 mM ARS solution to immobilize ARS in the film. The ARS-immobilized electrode was thoroughly rinsed in buffer solution (e.g., more than 12 h) before electrochemical measurements. Figure 3 shows the typical CV of the electrode after ARS immobilization. No redox peak was observed for the (PEI/CMC)_10_PEI film-coated electrode before ARS immobilization (data not shown). In contrast, well-defined redox peaks were observed in the potential range from −0.5 to −0.7 V, which originated from redox reactions of 9,10-anthraquinone moiety of ARS [16], confirming that the redox activity of ARS is preserved even in the LbL films. The amount of redox-active ARS in the (PEI/CMC)_10_PEI film was calculated to be 2.6 × 10^−9^ mol·cm^−2^ and 3.0 × 10^−9^ mol·cm^−2^ by integrating the peak area of the reduction and oxidation peaks, respectively, in the CV. The amount of redox-active ARS thus obtained is approximately 38–44% of the value obtained based on the UV-visible spectra (*vide supra*), suggesting a significant portion of ARS immobilized in the film is not involved in the redox reactions. Figure 4A shows the CVs recorded on the ARS-immobilized (PEI/CMC)_10_PEI film-coated electrode by changing the scan rate from 10 to 400 mV/s. The peak current of the CVs depended almost linearly on the square root of the scan rate (Figure 4B), suggesting that the redox reaction of ARS is diffusion-controlled [23]. Similar redox reactions of ARS on fluoropolymer-modified electrodes and anthraquinone sulfonate monolayer-modified electrodes have been reported [24,25]. Thus, the above results demonstrated that redox-active coatings can be prepared on the surface of an electrode by immobilizing ARS in the (PEI/CMC)_10_PEI film. 

It has been reported that the 1,2-dihydroxy group of ARS is also electrochemically active, though the reaction is irreversible due to the instability of the oxidation product [16]. In fact, we observed an oxidation peak at approximately 0.4 V in the CV for the ARS-immobilized electrodes (data not shown). However, the oxidation peak decreased during the repeated scanning of the electrode potential. Therefore, in this study, we focused on the redox reaction that occurred in the range from −0.5 to −0.7 V originating from the 9,10-anthraquinone moiety of ARS. 

### 3.3. Redox Properties of ARS-Immobilized PEI/CMC LbL Film in the Presence of PBA

**Figure 5:**
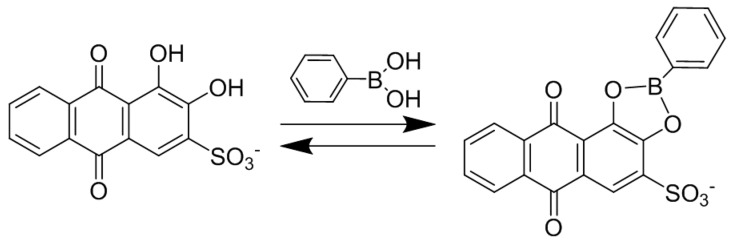
A binding equilibrium of ARS and PBA.

The redox behavior of ARS-immobilized electrodes may be modified in the presence of PBA in the solution, because PBA binds to the 1,2-dihydroxy group of ARS to form boronate ester (ARS-PBA) (Figure 5). In fact, it has been reported that ARS exhibits two oxidation peaks in the CV in solutions containing PBA [16,17,18,19]. One of the two oxidation peaks was ascribed to the oxidation reaction of ARS-PBA, while the other originated from free ARS. Therefore, it is interesting to evaluate the redox property of the ARS-immobilized (PEI/CMC)_10_PEI film-coated electrode in a PBA solution. 

**Figure 6:**
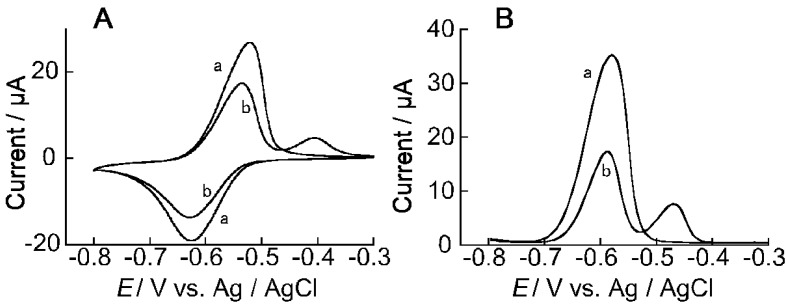
(**A**) CVs of the ARS-immobilized (PEI/CMC)_10_PEI film-coated Au electrode recorded in the absence (a) and presence of 1 mM PBA (b) in 10 mM HEPES buffer at pH 7.5. Scan rate, 50 mV s^−1^. (**B**) DPVs of the ARS-immobilized (PEI/CMC)_10_PEI film-coated Au electrode recorded in the absence (a) and presence of 1 mM PBA (b) in 10 mM HEPES buffer at pH 7.5.

Figure 6A shows the CV of the ARS-immobilized (PEI/CMC)_10_PEI film-coated electrode in the presence and absence of PBA. The CV exhibited two distinct oxidation peaks at −0.52 and −0.41 V in 1 mM PBA solution. The latter peak observed at −0.41 V may be ascribed to the oxidation of ARS-PBA formed in the film, while the former originated from free ARS, because PBA itself exhibits no redox reaction in this potential range. These results are basically in line with the redox reactions of ARS observed in aqueous solutions [16]. We have separately found that the intensity of the oxidation peak at −0.41 V increased with increasing the concentration of PBA added to the solution, supporting the hypothesis that the oxidation peak originated from ARS-PBA. Figure 6B shows the DPV of the ARS-immobilized (PEI/CMC)_10_PEI film-coated electrode in the presence and absence of PBA. The oxidation peak observed at −0.47 V is attributed to the oxidation reaction of ARS-PBA. 

**Figure 7:**
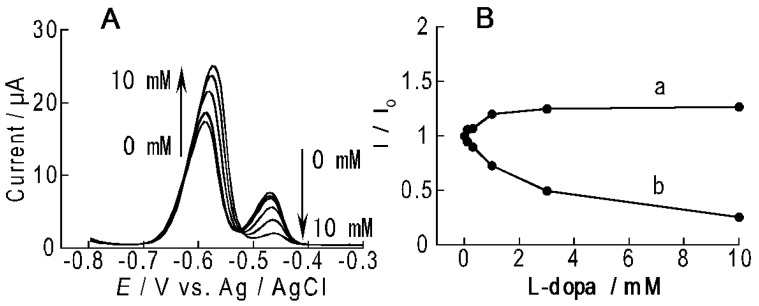
(**A**) DPVs of the ARS-immobilized (PEI/CMC)_10_PEI film-coated Au electrode in 1 mM PBA solution (10 mM HEPES buffer at pH 7.5) in the presence of 0–10 mM L-dopa; (**B**) Relative intensity of peak currents in DPVs at ca. −0.59 V (a) and ca. −0.47 V (b) as a function of the concentration of L-dopa. I denotes the peak current in the presence of L-dopa, while I_0_ is the peak current in the absence of L-dopa.

ARS-PBA formed in the LbL film may decompose in the presence of diol compounds as a result of competitive binding of diols to PBA [26]. The ARS-PBA content in the film would decrease upon adding diol compounds to the solution, resulting in decreased peak current for the reaction of ARS-PBA. Figure 7A shows DPVs of the ARS-immobilized (PEI/CMC)_10_PEI film-coated electrode in the absence and presence of 0.01–10 mM·L-dopa in the PBA solution. In the absence of L-dopa, the DPV provided two oxidation peaks ascribable to free ARS and ARS-PBA at −0.59 V and −0.47 V, respectively. The intensity of the oxidation peak at −0.47 V reduced with increasing the concentrations of L-dopa, while the oxidation peak at −0.59 V increased (Figure 7B). It is reasonable to assume that L-dopa binds to PBA in the film as well as forms L-dopa-PBA adduct in the solution, decreasing the concentration of ARS-PBA in the film. It is noted here that L-dopa itself shows an oxidation peak at 0.4–0.6 V, due to the oxidation reaction of the 3,4-dihydroxy group under similar experimental conditions [27,28]. Consequently, the oxidation peaks in the DPV can be fully ascribed to the oxidation reactions of ARS and ARS-PBA in the film. In other words, the ARS-immobilized electrode enables the electrochemical detection of L-dopa by recording voltammetric signals in the negative potential range, otherwise a higher positive potential has to be applied to oxidize L-dopa for the detection. The present system would be useful for the detection of other diol compounds as well, such as sugars, in view of the fact that PBA derivatives have been widely used in optical sensing of sugars [29,30]. 

## 4. Conclusions

The present study has demonstrated that ARS can be confined in the (PEI/CMC)_5_PEI and (PEI/CMC)_10_PEI films through electrostatic interactions to form redox-active surface coatings on an electrode. ARS binds PBA in the LbL film to form boronate ester ARS-PBA, providing an additional oxidation peak in its CV and DPV. The intensity of the oxidation peak arising from ARS-PBA decreased upon adding L-dopa to the solution, depending on the concentration, suggesting a potential use of the ARS-confined film-coated electrodes for the voltammetric detection of L-dopa.
